# Influence of hemostatic agents upon the outcome of periapical surgery: Dressings with anesthetic and vasoconstrictor or aluminum chloride

**DOI:** 10.4317/medoral.18002

**Published:** 2012-12-10

**Authors:** María Peñarrocha-Diago, Laura Maestre-Ferrín, David Peñarrocha-Oltra, Thomas von Arx, Miguel Peñarrocha-Diago

**Affiliations:** 1 Associate Professor of Oral Surgery. Valencia University Medical and Dental School. Valencia, Spain; 2 Master in Oral Surgery and Implantology. Valencia University Medical and Dental School. Valencia, Spain; 3 Resident of the Master in Oral Surgery and Implantology. Valencia University Medical and Dental School. Valencia, Spain; 4 Vice Chairman and Associate Professor. Department of Oral Surgery and Stomatology. School of Dental Medicine, University of Bern. Switzerland; 5 Chairman of Oral Surgery. Director of the Master in Oral Surgery and Implantology. Valencia University Medical and Dental School. Valencia, Spain. Investigator of the IDIBELL Institute

## Abstract

Objective: To evaluate the effects of different hemostatic agents upon the outcome of periapical surgery. Design: A retrospective study was made of patients subjected to periapical surgery between 2006-2009 with the ultrasound technique and using MTA as retrograde filler material. We included patients with a minimum follow-up of 12 months, divided into two groups according to the hemostatic agent used: A) dressings impregnated in anesthetic solution with adrenalin; or B) aluminum chloride paste (Expasyl™). Radiological controls were made after 6 and 12 months, and on the last visit. The global evolution scale proposed by von Arx and Kurt (1999) was used to establish the outcome of periapical surgery. Results: A total of 96 patients (42 males and 54 females) with a mean age of 40.7 years were included. There were 50 patients in the aluminum chloride group and 46 patients in the anesthetic solution with vasoconstrictor group. No significant differences were observed between the two groups in terms of outcome after 12 months - the success rate being 58.6% and 61.7% in the anesthetic solution with vasoconstrictor and aluminum chloride groups, respectively (p>0.05). Conclusion: The outcome after 12 months of follow-up was better in the aluminum chloride group than in the anesthetic solution with vasoconstrictor group, though the difference was not significant.

** Key words:**Aluminum chloride, bleeding control, hemostasis, periapical surgery, outcome.

## Introduction

Many factors can influence the outcome of periapical surgery. In this context, adequate bleeding control is essential for the success of periapical surgery, since it improves visualization of the surgical site, minimizes the operating time, and is a requirement for the insertion of most retrograde filling materials (1,2).

Different agents and techniques have been used to secure hemostasis in periapical surgery. Bone wax has been used for many years and is easy to handle, though remaining traces of this material can cause adverse tissue reactions (3). Similar problems have been observed with ferric sulfate: when not completely eliminated from the surgical site, it gives rise to foreign body reactions that complicate healing (4). Vickers et al. (5) evaluated the hemostatic efficacy and cardiovascular effects of ferric sulfate and of cotton pellets impregnated with racemic adrenaline. Both agents afforded surgical hemostasis, and there were no significant changes in the systemic cardiovascular parameters with either of them. In a similar study, Vy et al. (6) concluded that collagen sponges impregnated with adrenaline affords excellent bleeding control, with no evident changes in either blood pressure or heart rate.

Von Arx et al. (7) introduced the use of Expasyl™ (Pierre Rolland, Merignac, France) for securing hemostasis in periapical surgery. This is a paste containing aluminum chloride and kaolin, and is normally used to produce gingival retraction (8,9). In an experimental study, von Arx et al. (7) compared the hemostatic efficacy and the tissue reactions of bone wax, ferric sulfate, aluminum chloride, and a combination of aluminum chloride and ferric sulfate. Expasyl™ alone or in combination with ferric sulfate was found to be the most effective agent, and the inflammatory reactions were limited to the bone defects, with no spread to the surrounding tissues. Jensen et al. (10) used the same study design to compare the effects of 5 hemostatic techniques: Expasyl™ + Stasis®, Expasyl™ + Stasis® + bone crypt freshening with a drill, Spongostan®, Spongostan® + adrenaline, and electrocautery. The most effective methods for reducing bleeding were Expasyl™ + Stasis® and electrocautery, but adverse tissue reactions were observed (necrotic bone, inflammatory cells, absence of bone repair). Such tissue damage was not recorded when the superficial bone layer was eliminated with rotary instruments.

A number of studies have been published involving different materials used to secure hemostasis in periapical surgery, though none have correlated the outcome of periapical surgery to the hemostatic agent used.

The present study was carried out to compare the outcome of periapical surgery when using two different hemostatic agents: dressings impregnated with anesthetic solution containing a vasoconstrictor (adrenaline), and aluminum chloride paste (Expasyl™).

## Material and Methods

-Sample selection

A retrospective study was carried out in the Oral Surgery Unit (Valencia University Medical and Dental School, Valencia, Spain) covering the period between October 2006 and March 2009 and including the patients subjected to periapical surgery with the ultrasound technique, using MTA (ProRoot®, Dentsply, USA) as retrograde filling material. The study was approved by the local Ethics Committee, and all patients gave informed consent to participation in the study.

In all cases dressings impregnated in anesthetic solution with vasoconstrictor, or aluminum chloride paste, were used for securing hemostasis, and the minimum follow-up period was 12 months. Specifically, we used dressings impregnated in anesthetic solution with vasoconstrictor (4% articaine with adrenaline 1:100,000)(Inibsa, Lliça de Vall, Barcelona, Spain) in the patients treated between October 2006 and December 2007, and aluminum chloride (Expasyl™, Produits Dentaires Pierre Rolland, Merignac, France) in the patients treated between January 2008 to March 2009.

In the patients with periapical lesions measuring over 20 mm in diameter, with tunnel lesions rupturing the vestibular and lingual cortical layers, or with apicomarginal defects with complete vestibular cortical loss, hemostasis was secured with dressings impregnated in anesthetic solution with vasoconstrictor, due to the difficulty of eliminating the remaining traces of Expasyl™ in such situations. In these cases we always used bone regeneration techniques, and decided to exclude the patients from the study, since they all belonged to the adrenalin with vasoconstrictor group.

We likewise excluded those patients in which no hemostasis material had been used (9 patients). The resulting initial study sample consisted of 123 patients, divided into two groups according to the hemostatic agent used: A) dressings impregnated in anesthetic solution with adrenalin (55 patients); or B) aluminum chloride paste (Expasyl™)(68 patients).

Eight patients were excluded due to incomplete study protocols (one in the dressings group and 7 in the Expasyl™ group), 14 because of a lack of follow-up (3 in the dressings group and 11 in the Expasyl™ group), and 5 patients in the dressings group in which bone regeneration techniques had been used (3 patients with periapical lesions measuring over 20 mm in diameter, one with a periapical lesion rupturing the vestibular and lingual cortical layers, and one patient with an apicomarginal defect and complete vestibular cortical loss).

The final study sample thus consisted of 96 patients: 46 belonging to the dressings with anesthetic and vasoconstrictor group and 50 to the Expasyl™ group.

-Surgical technique

**Figure 1:**
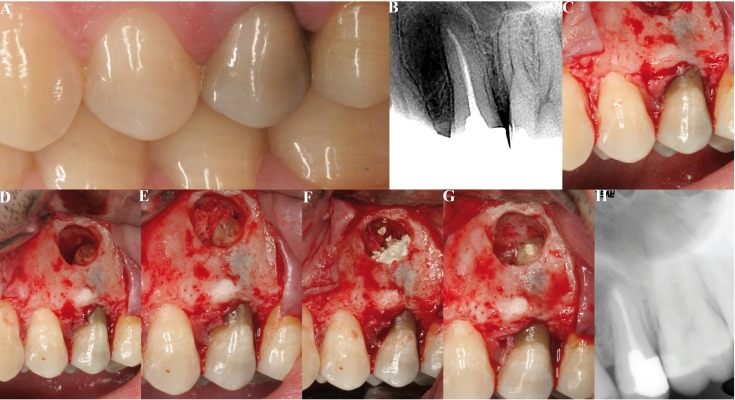
Periapical surgery of an upper second premolar using dressings impregnated with anesthetic and vasoconstrictor as hemostatic agent. A) Preoperative clinical view. B) Periapical X-ray view showing a periapical radiotransparent area in relation to the apex of the second premolar. C) Intraoperative view following ostectomy. D) View of the bone cavity after curettage of the periapical lesion. E) Hemostasis with dressing impregnated with anesthetic and vasoconstrictor. F) Retrograde filling with MTA. G) View of retrograde filling after eliminating the excess MTA and impregnated dressing. H) Radiological control after 12 months of follow-up.

**Figure 2:**
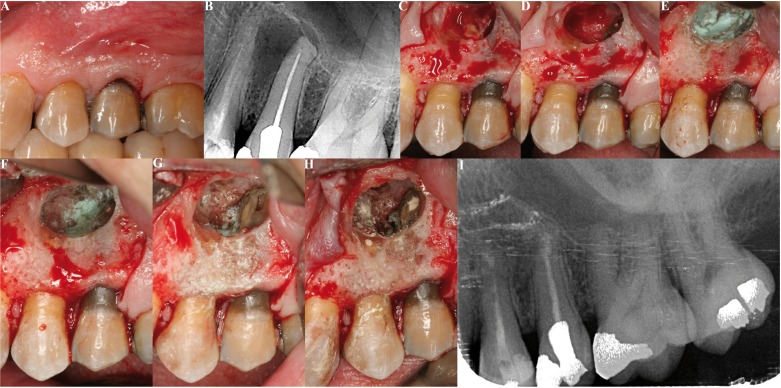
Upper left premolars periapical surgery using Expasyl™ to control bleeding. A) Preoperative clinical view. B) Periapical X-ray view showing a periapical radiotransparent area between the apexes of both premolars. C) Periapical lesion after ostectomy. D) View of the bone cavity after curettage of the periapical lesion. E) Expasyl™ inserted in the bone crypt and allowed to act during two minutes. F) The cavity is irrigated with sterile saline solution to remove the excess Expasyl™. G) Retrograde cavities prepared in both teeth. H) Retrograde filling with MTA. I) Periapical radiological control after 12 months of follow-up.

All the operations were made by the same surgeon (MPD). Locoregional and infiltrating anesthetic techniques were used with 4% articaine and adrenaline 1:100,000 (Inibsa, Lliça de Vall, Barcelona, Spain). Intrasulcular incisions were made with one or two vertical releasing incisions for raising triangular or trapezoidal mucoperiosteal flaps. Ostectomy was performed using a rounded 0.27 mm tungsten carbide drill (Jota, Switzerland) under abundant irrigation with saline solution. The minimum apical resection needed to access the apexes was made, followed by apical curettage. The cavity was prepared for retrograde filling using a Piezon Master® 400 ultrasound instrument (EMS, Electro Medical Systems, S.A., Switzerland) at full power with diamond-surfaced ultrasound tips for periapical surgery. To facilitate the procedure, use was made of a Medi Pack Pal endoscope (Farol Store and Co., Tuttlingen, Germany) and Moeller® Dental 300 surgical microscope (Möller-Wedel International, Bedel, Germany). Bone cavity drying and bleeding control was carried out in group A by means of small sterile dressings impregnated with anesthetic solution with adrenaline as vasoconstrictor at a concentration of 1:100,000 (Inibsa, Lliça de Vall, Barcelona, Spain), placed within the bone crypt. Dry dressings were then placed and pressure was applied for two minutes, after which all the dressings except the first impregnated dressing were removed, and the surgical procedure was continued – removing the impregnated dressing before saline irrigation and suturing (Fig. 1). In group B use was made of ExpasylTM (Expasyl, Produits Dentaires Pierre Rolland, Merignac, France) applied during two minutes (Fig. 2). Finally, ProRoot® (Dentsply, USA) mineral trioxide aggregate (MTA) retrograde filling material was prepared, inserted and condensed, following the indications of the manufacturer. In the patients in which Expasyl™ was used, the bone crypt was refreshed with a rounded drill under abundant irrigation before suturing. Suturing was carried out with Tevdek® non-reabsorbable sutures (Deknatel®, Teleflex®, Athlone, Ireland) composed of polyester fibers coated with polytetrafluoroethylene (PTFE).

In all cases the following medication was prescribed in the week after surgery: amoxicillin 500 mg with clavulanic acid 125 mg every 8 hours during 6 days; ibuprofen 600 mg every 8 hours during 4 days; 0.12% chlorhexidine gluconate rinses three times a day during 7 days; and paracetamol 500 mg upon demand in the event of important pain.

-Radiographic evaluation

Periapical and panoramic X-rays were obtained with an XMIND® intraoral system (Groupe Satelec - Pierre Rolland®, France), together with digital panoramic X-rays (OP100®, Instrumentarium Imaging, Tuusula, Finland).

Radiographic controls were made immediately after surgery, after 6 and 12 months, and on occasion of the subsequent annual control visits. The months elapsed from surgery to final control (counting to the last visit of each patient) or until failure were recorded for calculating mean patient follow-up.

-Scales of success

The outcome of periapical surgery was assessed based on the global evolution scale proposed by von Arx and Kurt (11), which combines clinical and radiological criteria. Outcome was classified as: 1) success: when bone regeneration was ≥ 90% and the clinical and pain score was 0 (on a scale of 0-3); 2) improvement: when bone regeneration was 50-90% and the clinical and pain score was 0; and 3) failure: when bone regeneration was < 50% or there were clinical symptoms.

-Data collection and statistical analysis

A clinical history was compiled for all patients, following a previously established protocol - recording the personal antecedents of interest, collecting all the clinical and radiographic data of the patients in detail, and registering the pre-, intra- and postoperative characteristics.

The SSPS version 15 statistical package for MS Windows was used for analyzing the data. In each study group we performed a descriptive analysis of all the variables (gender, age and area of the periapical lesions), checking that there were no differences between the two groups for any of these variables. The outcomes of periapical surgery after 6 and 12 months and at the final control, in each group, were compared using the chi-squared test, with a statistical significance level of p<0.05. In all cases the necessary tests for confirming the applicability of the different statistical techniques were carried out.

## Results

The study comprised a total of 96 patients (42 males and 54 females) with a mean age of 40.7 years (range 12-67). The Expasyl™ group consisted of 50 patients (with 81 apicoectomized teeth, 110 treated roots and 125 treated canals), while the anesthetic solution with vasoconstrictor group comprised 46 patients (with 58 apicoectomized teeth, 78 treated roots and 91 treated canals). The mean duration of follow-up was 17.1 months (range 1-41). In the anesthetic solution with vasoconstrictor group the mean duration of follow-up was 18.6 months (range 1-41), versus 16.6 months (range 1-26 months) in the Expasyl™ group. There were no significant differences in the duration of follow-up between the two groups of patients (t = 1.12; p = 0.26).

 Table 1 shows the mean age and gender distribution of the patients in each group, together with the data referred to the area of the periapical lesions. There were no significant differences between the two groups in terms of mean age, gender distribution or periapical lesion size.

**Table 1:**
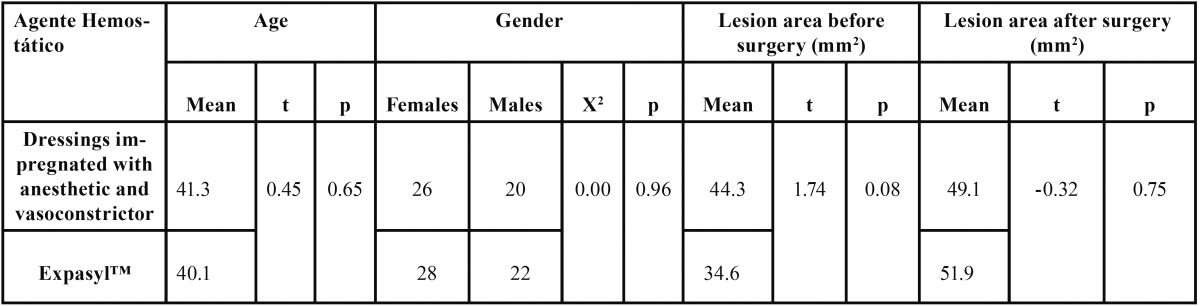
Mean age, gender distribution and periapical lesion size in each group.

The global outcome in each group after 6 and 12 months and on occasion of the final control, as established from the global evolution scale of von Arx and Kurt (11), is reported in  Table 2. The periapical surgery success rate at last control was 62.5% in the Expasyl™ group and 58.6% in the group treated with dressings impregnated with anesthetic solution and vasoconstrictor. The outcome of periapical surgery was better in the Expasyl™ group than in the anesthetic solution with vasoconstrictor group after 12 months of follow-up and on occasion of the final control, though the differences were not statistically significant.

**Table 2:**
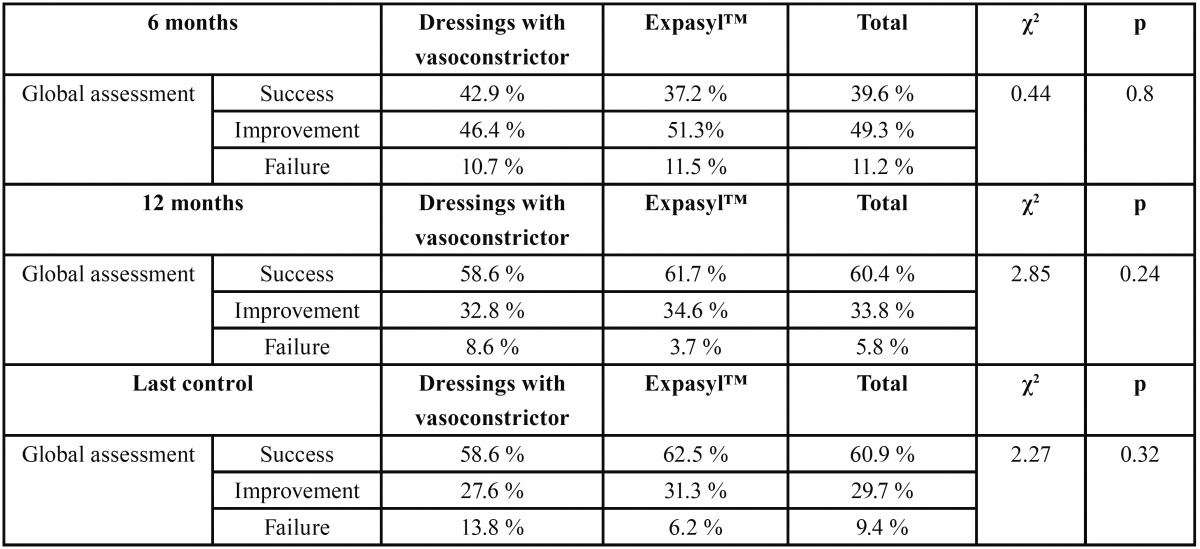
Outcome of periapical surgery after 6 and 12 months, and at the last control visit, according to the criteria of von Arx and Kurt (11).

## Discussion

The outcome of periapical surgery can differ considerably from one study to another, depending on the patient inclusion criteria used, the scales employed to score success, and the duration of follow-up (12). In the present study we used the criteria of von Arx and Kurt for assessing the outcome of periapical surgery (11). According to Peñarrocha et al. (13) and Ortega-Sanchez et al. (14), this is the scale offering the best information, and which agrees best with the rest of the scales. The minimum duration of follow-up needed to determine the outcome of periapical surgery has been established as 12 months (15-17).

On occasion of the control visit after 12 months, 60.4% of the teeth showed treatment success, 33.8% improvement, and 5.8% failure. At the final control (after an average of 17.4 months of follow-up), 60.9% of the teeth showed treatment success, 29.7% improvement, and 9.4% failure. The success rates reported by other authors also using ultrasound and MTA, independently of the hemostatic material used, range from 74-92% (18-24), though the study designs and success scoring criteria differ among the publications. Most of them (18-22,24) followed the criteria established by Rud et al. (16) and Molven et al. (25), calculating the success rates by combining the number of fully healed teeth and incompletely healed teeth (scar), and regarding them all as healed cases. In order to compare our results with those of the above authors, we summed the percentages of success and improvement to obtain a healing rate after 12 months of 94.2%, which falls within the described ranges. Tsesis et al. (26) published a metaanalysis quantifying the outcomes of periapical surgery and studying the influence of different factors upon treatment outcome. These authors obtained a periapical surgery success rate after one year of follow-up of 91.6%, which coincides with our own findings and with those of von Arx et al. in their metaanalysis (91.4% for teeth filled with MTA)(27).

Only one study to date (23) has correlated the outcome of periapical surgery to the use of hemostatic agents. In order to control bleeding of the bone crypt, the authors used dressings impregnated with adrenaline or ferric sulfate. The evaluation was made according to whether either material had been used or not, without differentiating the results obtained with each of them individu-ally. The authors found that the use of a hemostatic agent is not predictive of the outcome of periapical surgery. Von Arx et al. (20) used aluminum chloride (Expasyl™) and/or ferric sulfate to secure hemostasis during periapical surgery of 194 human teeth, with a success rate of 90.2%, though the influence of the hemostatic agent upon the outcome of surgery was not analyzed.

In our study we found no significant differences in the outcome of periapical surgery between the two groups. In the control performed after 6 months, the outcome was seen to be poorer in the Expasyl™ group, though not significantly so, while after 12 months the opposite situation was observed (i.e., outcome was comparatively better in the Expasyl™ group). The evolution between 6 and 12 months after surgery was more favorable in the Expasyl™ group than in the group treated with dressings impregnated with anesthetic solution and vasoconstrictor. This could be explained by the presence of remaining traces of Expasyl™ - despite having favored bleeding within the bone cavity - which could have delayed bone formation during the first months. However, the more positive course seen in this same group over the subsequent months was probably due to better quality retrograde fillings resulting from the excellent field dryness and visibility conditions afforded by Expasyl™, and which would minimize bacterial filtration between the periapex and root canal system. Nevertheless, since patient assignment to each group was not randomized (this being a retrospective study), the comparatively more favorable results obtained in the Expasyl™ group may have been a mere coincidence or could have been influenced by the surgeon learning curve. Randomized, controlled prospective studies involving large sample sizes are therefore needed to more firmly establish the effects of Expasyl™ upon the outcome of periapical surgery. The healing percentages (summing success and improvement) after 12 months were 96.3% in the Expasyl™ group and 91.4% in the group treated with dressings impregnated with anesthetic solution and vasoconstrictor. These figures are slightly higher than the 90.2% healing rate mentioned above, corresponding to the study of von Arx et al. (20) using Expasyl™ and/or ferric sulfate to control bleeding.

It has been seen that aluminum chloride produces inflammatory reactions in the soft tissues when used for gingival retraction (28,29). In the study of von Arx et al. (7) in rabbits, Expasyl™ alone or in combination with ferric sulfate induced foreign body reactions with evidence of an inflammatory tissue response three and 12 weeks after placement. Moreover, in contrast to the control sites, bone formation was minimal and delayed. The authors therefore recommended cleaning the surgical wound with a curette and refreshing the bone walls with a rounded drill before wound closure. Jensen et al. (10) noted that adverse reactions to Expasyl™ did not occur if the traces of the paste were eliminated from the bone crypt with rotary instruments. In our study we favored bleeding within the bone cavity before suturing, and the teeth in the Expasyl™ group had a better prognosis than those belonging to the anesthetic solution with vasoconstrictor group after 12 months of follow-up and on occasion of the last control visit (non significant differences) – though not so at the first control after 6 months. It is therefore possible that foreign body reactions to the paste were not entirely avoided by our procedure, resulting in slower healing in the Expasyl™ group in the first few months. Further studies are needed to determine whether the surgical improvements and facilities afforded by Expasyl™ effectively contribute to improve the outcome of periapical surgery over the long term.

## Conclusion

The outcome after 12 months of follow-up was better in the Expasyl™ group than in the anesthetic solution with vasoconstrictor group, though the difference was not significant.
